# Cigarettes Butts and the Case for an Environmental Policy on Hazardous Cigarette Waste

**DOI:** 10.3390/ijerph6051691

**Published:** 2009-05-20

**Authors:** Thomas E. Novotny, Kristen Lum, Elizabeth Smith, Vivian Wang, Richard Barnes

**Affiliations:** 1 Center for Tobacco Control Research and Education University of California San Francisco, San Francisco, CA, 94143, USA; E-Mails: kristen.lum@ucsf.edu (K.L.); Libby.Smith@ucsf.edu (E.S.); Vivian.wang@ucsf.edu (V.W.); Richard.barnes@ucsf.edu (R.B.); 2 Graduate School of Public Health, San Diego State University, San Diego, CA 92186, USA

**Keywords:** cigarette litter, waste, butts, smoking, filters, environment

## Abstract

Discarded cigarette butts are a form of non-biodegradable litter. Carried as runoff from streets to drains, to rivers, and ultimately to the ocean and its beaches, cigarette filters are the single most collected item in international beach cleanups each year. They are an environmental blight on streets, sidewalks, and other open areas. Rather than being a protective health device, cigarette filters are primarily a marketing tool to help sell ‘safe’ cigarettes. They are perceived by much of the public (especially current smokers) to reduce the health risks of smoking through technology. Filters have reduced the machine-measured yield of tar and nicotine from burning cigarettes, but there is controversy as to whether this has correspondingly reduced the disease burden of smoking to the population. Filters actually may serve to sustain smoking by making it seem less urgent for smokers to quit and easier for children to initiate smoking because of reduced irritation from early experimentation. Several options are available to reduce the environmental impact of cigarette butt waste, including developing biodegradable filters, increasing fines and penalties for littering butts, monetary deposits on filters, increasing availability of butt receptacles, and expanded public education. It may even be possible to ban the sale of filtered cigarettes altogether on the basis of their adverse environmental impact. This option may be attractive in coastal regions where beaches accumulate butt waste and where smoking indoors is increasingly prohibited. Additional research is needed on the various policy options, including behavioral research on the impact of banning the sale of filtered cigarettes altogether.

## 1. The History and Function of Cigarette Filters

**Figure 1.**
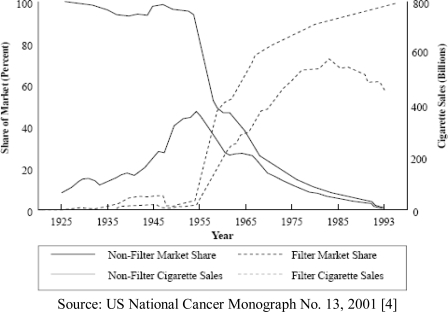
Market share and sales of filtered and non-filtered cigarettes in the United States, 1925–1993.

The cellulose-acetate filter was added to cigarettes in the 1950s in the wake of increasingly convincing scientific evidence that cigarettes caused lung cancer and other serious diseases [1]. Filters were found to reduce the machine-measured yields of tar and nicotine in smoked cigarettes, and at first this seemed to be a healthy technological improvement in the cigarette product. In 1966, a review by the US Public Health Service concluded that, “The preponderance of scientific evidence strongly suggests that the lower the ‘tar’ and nicotine content of cigarette smoke, the less harmful would be the effect.” Following this report, both Government and tobacco industry scientists conducted studies of cigarette manufacturing and tobacco cultivation that could lead to lower “tar” and nicotine yields. Cigarette manufacturers promoted such products, especially filtered cigarettes, through advertising that included an implied health claim for ‘safer’ cigarettes. Some epidemiological studies have alluded to reduced health impacts attributable to lower tar- and nicotine-yielding cigarettes [2,3]; in fact, the sales-weighted averages of these constituents in cigarettes has dramatically declined over the last 50 years. Nevertheless, smokers who switched to these low-yield brands did not substantially alter their exposure to tar and nicotine because of compensatory smoking (deeper and more frequent puffing, plugging ventilation holes on filters, etc.) and the changes in the way cigarettes were manufactured. To address this confusion, the National Cancer Institute undertook a comprehensive review of low-tar and low-nicotine yielding cigarettes’ potential health benefits. Its 2001 *Monograph 13, Risks Associated with Smoking Cigarettes with Low Machine-Measured Yields of Tar and Nicotine,* [4] concluded that “Epidemiological and other scientific evidence, including patterns of mortality from smoking-caused diseases, does not indicate a benefit to public health from changes in cigarette design and manufacturing over the last fifty years.” In addition, a 2006 US Department of Justice ruling against the tobacco companies, at present stayed and pending appeal, “bans terms including “low tar,” “light,” “ultra light,” “mild,” and “natural” that have been used to mislead consumers about the health risks of smoking and prohibits the tobacco companies from conveying any explicit or implicit health message for any cigarette brand” [5]. Over the last 50 years, smokers switched almost entirely (99%) to filtered cigarettes (Figure 1), and nearly all of these sold in the United States are made of cellulose acetate, a plastic product [6].

Filters likely discourage many smokers from making the quit attempt because they still cling to the belief that filtered cigarettes are protective of their health; thus, filters may have overall a detrimental effect on population health. Filters are a rod of about 12,000 fibers, and fragments of this material become separated from the filter during the manufacturing process and may be released during inhalation of a cigarette. It has been reported in tests on 12 popular brands that fibers are inhaled and also ingested, and filter fibers have been reportedly found in the lung tissue of patients with lung cancer [7]. Furthermore, consumer preference for filtered cigarettes may have been associated with a histological shift in predominant lung cancer type from squamous cell to the more aggressive adenocarcinoma cell type [8].

Currently, cigarette manufacturers are contemplating and test marketing additional “reduced harm” products, including new types of filters that may reduce toxic constituents in cigarette smoke (these new filters also contain cellulose acetate as well as new filter materials) [9]. Nonetheless, filters continue to be primarily a marketing tool to help sell cigarettes.

## 2. The Environmental Problem of Cigarette Butts

Whatever their direct health impact on or benefit to smokers, cigarette filters pose a serious litter and toxic waste disposal problem. Cellulose acetate is photodegradable but not bio-degradable. Although ultraviolet rays from the sun will eventually break the filter into smaller pieces under ideal environmental conditions, the source material never disappears; it essentially becomes diluted in water or soil [10,11].

While the environmental impact of a single disposed cigarette filter is minimal, there were 1.35 trillion filtered cigarettes manufactured in the United States in 2007, and of these, more than 360 billion were consumed here [12]. About 680,000 tons of cellulose acetate was used in the production of these filtered cigarettes. With 5.6 trillion filtered cigarettes consumed worldwide in 2002, and nine trillion expected by 2025, the global environmental burden of cigarette filters is also significant [13]. It is estimated that 1.69 billion pounds (845,000 tons) of butts wind up as litter worldwide per year [14].

**Figure 2.**
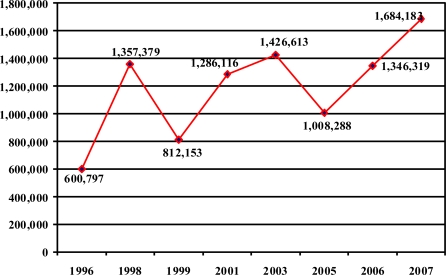
Cigarettes and Cigarette Filters Collected in the United States in the International Coastal Cleanup, 1996–2007. Source: Ocean Conservancy 2007.

Most attention has been given to the cigarette butt waste problem because of the filters that end up on beaches. The annual Ocean Conservancy’s International Coastal Cleanup (ICC) reports that ‘cigarette butts have been the single most recovered item since collections began’ [15]. Although volunteers collected 1,684,183 cigarette butts (33.6% of all debris) in the 2007 US Cleanup (Figure 2), these data likely underestimate total discarded filters. For example, a comprehensive cleanup in Orange County, California, yielded 20 times more butts than the estimated ICC total for that beach for the same year [16].

The cigarette butts recovered from beaches are not necessarily due to cigarettes that are smoked on them. Butts are dropped on sidewalks or thrown from moving cars; they then move to the street drains, and thus to streams, rivers, and the oceans. In addition, since the early 1980s there has been increasing concern about the health consequences of passive smoking, and thus more smoking occurs outdoors, likely contributing to this chain of events. As a consequence, cigarette butts become unsightly and difficult-to-remove waste in multiple locations, including streets, storm drains, streams, and beaches. In a review of litter cleanup project reports, the *Keep America Beautiful Campaign* reported that cigarette butts comprise from 25 to 50 percent of all collected litter items from roadways and streets. One report from a college campus estimated the cost of cigarette litter cleanup at $150,000 for a single, two-week-long effort. No other economic impact studies have been reported [17]. Their non-biodegradability means that they also increase landfill demands, add costs to municipalities’ waste disposal programs, and create environmental blight in public spaces.

Discarded cigarette butts are not only unsightly; they are also toxic in and of themselves. Environmental groups have expressed concern for marine creatures that ingest littered filters [18,19]. A 2006 laboratory study found that cigarette butts were found to be acutely toxic to a freshwater cladoceran organism and a marine bacteria (microtox) and that the main cause of toxicity was attributed to nicotine and ethylphenol in the leachates from cigarette butts [20]. A 1997 report from the Rhode Island Department of Health reported 146 cases of cigarette butt ingestion among children < 6 years old; of these, approximately one-third displayed transient nicotine toxicity [21]. Even if properly disposed, cigarette butts are hazardous solid waste. It is unknown as to how many must be consumed to cause adverse health effects in marine animals such as birds or mammals.

## 3. The Tobacco Industry Response

In the 1990s, market research prompted cigarette manufacturers to recognize that environmental concerns about discarded butts might become more important to consumers and policymakers. A 1992 Philip Morris USA internal memo identified cellulose acetate filters as non-degradable material and reported that Eastman Chemical Products Company and Celanese Fibers Company were conducting research on cellulose acetate degradation [22]. Alternatives to the cellulose acetate filter were also pursued by Brown & Williamson Tobacco Company [23] and RJR, whose ‘Degradable Team’ reported in the minutes from an April 4, 1996, meeting that it had tested five biodegradable filter prototypes in sensory evaluation tests. However, these filters were found to be unacceptable to smokers: “all products had greater artificial lit aroma, less tobacco taste, more artificial taste, more generic taste, less sweet, more bitter, less tobacco aftertaste, greater bitter, non-tobacco aftertaste and greater drying.”[24]. In 1998, RJR scientists filed a US patent on a “degradable smoking article” that utilized dissociable cigarette parts to accelerate disintegration by increasing exposure of surface areas to “natural elements”. However, their research found that the disintegrated filter components were still deposited in the environment as small particles [25].

CORESTA, the tobacco industry’s international research organization, formed a ‘Cigarette Butt Degradability Task Force’ in the early 1990s to “develop a test to determine the rate of degradability of a complete cigarette butt” [26]. The task force’s membership of cigarette makers, filter suppliers, paper manufacturers, and adhesive companies displayed extensive interest in biodegradability research. If a biodegradable filter were marketable, these industries would reap significant financial benefits through a new marketing tool that would help smokers identify themselves as environmentally friendly. However, the task force’s final report stated that their objective “was made more difficult by the fact that most of the available reference work supported efforts to enhance stability not degradability, and were applied to single component products, not systems composed of different types of materials”. The task force disbanded in 2000 after CORESTA found that it was “unlikely that the level of interest could justify the scale of the effort”, which would require more data collection and the development of instrumentation to establish a standardized test for cigarette filter degradation [27].

In 2000, Philip Morris’ consumer research on cigarette litter found that the issue was not “top of mind” for smokers, that there is ritualized behavior in the disposal of cigarette butts, and that “adults who choose to smoke need convenient alternatives to cigarette disposal” [28]. As a result of this research, Philip Morris proposed distribution of convenient disposal receptacles and direct communication with smokers to encourage them to dispose of butts in an environmentally conscious manner. Subsequently, Phillip Morris became one of the major supporters of the *Keep America Beautiful Campaign* ([KAB] a non-profit, grass roots organization), which encourages individual responsibility for proper butt disposal and other wastes [29]. However, there are no evaluation data on the effectiveness of such campaigns in reducing butt litter. It may be that Philip Morris’ interests lie primarily in shifting the responsibility for butt waste to the consumer; KAB’s efforts focus on public education and increasing availability of butt receptacles, including hand held ashtrays; its campaigns support Philip Morris’ corporate social image [30]. In 2007, it received a $3 million grant from Philip Morris USA for its butt litter campaigns [31].

The tobacco industry has considered this problem further with some of their own research on filter degradability. Philip Morris documents described “Project Natural” at the 1990 Philip Morris International Marketing Meeting, where the litter issue and the problems with filter degradability were discussed. The presenter stated: “to avoid this problem, the simplest solution would be to eliminate the filter! But this of course would defy consumer preference and make it difficult to control tar and nicotine levels” [32].

In a 2006 Stakeholder analysis and response project, RJR described these internal and industry-sponsored programs as mainly to develop test methods that define the photo, water and biological degradability of existing and new materials. RJRs final message to stakeholders was, “Our opinion is that the *current state of the art in material technology has not produced a material that is commercially feasible*. While some degradable materials have been identified, they are unsuitable because of poor taste, short shelf-life and physical instability during smoking, manufacturability and/or material variability. The company is continuing to look at all technological solutions to biodegradability” (emphasis added) [33].

Currently, there is no evidence that the industry has developed a marketable, degradable filter. However, one biotech company (Stanelco) has developed a food-starch-based filter and has appointed Rothschild International, to develop and test this device for possible widespread adoption [34]. Starch used in the filter is essentially a carbohydrate polymer found in foods such as potato and rice. The biodegradability of such filters could theoretically reduce the environmental impact of butt waste by being compostable. Stanelco has touted this filter as not only eco-friendly but 30 to 50% cheaper than cellulose acetate filters at bulk prices. Compared with cellulose acetate filters, the company claims that starch-based filters may also have health effects because smokers will not be exposed to “fall-out” of cellulose acetate fragments entering the lung through inhalation [35]. Even with starch-based composition, these filters may take two months to biodegrade, and they would still release toxic filtrates into the environment when they do so.

## 4. Community and State Response

In response to the issue of cigarette butt litter, some municipalities have banned smoking on beaches, including in Chicago, San Diego, and other areas (Table 1). These bans are widely seen as a good first step to controlling butt waste, but because of the runoff from streets to waterways to ocean, they will not eliminate them from beaches. Butts despoil these heavily used public spaces, which then become the responsibility of the state and local authorities to clean up. In California, a law that would ban smoking on all 64 state-run beaches and State Parks in California failed by two votes in 2004 in the state Senate and is currently under consideration again [36]. There appears to be considerable interest in beach smoking bans, mainly at the local level, where responsibility for cleanup resides. Detailed cost analyses and impact assessments on such bans are as yet lacking.

## 5. Policy Options to Reduce the Environmental Impact of Cigarette Butt Litter

Our previous report [37] established the environmental externalities of smoking, focusing on the enormous number of butts reported in international beach cleanups and on the hazardous wastes resulting from cigarette manufacturing processes. There is precedent for enacting state and local regulation to protect the environment from non-biodegradable solid waste from consumer products; we suggest several models for possible action against cigarette butt waste.

### 5.1. Labeling

Some products carry warnings printed on them advising consumers not to litter the packages or the product (aluminum cans, bottles, plastics, etc). This has never been proposed as a means of warning smokers about the non-biodegradability of filters (or of package litter). A warning label of sufficient size could be required as part of the proposed FDA regulatory authorization that simply states: “Cigarette filters are non-biodegradable hazardous waste. Disposal of filters should be in accordance with state law” (with appropriate state law requirements included on each package sold in the each state). These could go on to describe potential human toxicity, methods for safe handling, etc.

### 5.2. Deposit/Return

In the 1970s, Oregon and several other states introduced “bottle bills” as a way to reduce the hazards, clean-up costs, and waste of discarded glass containers (mostly from beverages). Deposit/recycling laws have been implemented around the world, in fact. These laws mandate that consumers pay a deposit when they purchase specified items which will be returned when the container is returned. The Oregon law is credited with reducing litter and increasing container recycling, with return rates of up to 90%. The Oregon Department of Environmental quality reports that discarded items covered by the laws were reduced from 40% of roadside litter collected to 6% [38]. In South Australia, there has been similar success with bottle bills and electronics [39]. Similarly, cigarettes could be sold with a “butt deposit” to be refunded when the pack is returned to the vender with the butts. As with bottles and cans, this could spark both more care on the part of smokers and provide income to others who retrieve any butts that smokers discard. It would also increase the opportunity costs of smoking, thus perhaps having a salutary effect on reduced cigarette consumption.

### 5.3. Waste Tax

Concern about toxic waste resulting from technology products such as computers, telephones, and televisions, has given rise to legislation implementing a consumer funded Advanced Recycling Fee (ARF); this is assessed at the point of purchasing electronic products [40]. These fees are intended to pay for the costs of recycling the item and disposing properly of any non-recyclable material. The fees are minimal (compared to the cost of the products), ranging from $6 to $10. Of note, this system functions with complete support of the manufacturers themselves, with core principals calling for shared responsibility. Adding a waste *fee* to cigarettes is another possibility, and the funds collected could be used to mitigate environmental consequences and to fund research on butt waste. A fee or tax has the added advantage of increasing costs of cigarettes, thereby reducing consumption. Such fees would have to be supported by careful litter audits and economic costs of cleanup studies.

### 5.4. Litigation

To date, most litigation against the tobacco industry has focused on the health costs that others (individuals, insurance companies, states) end up paying as a result of cigarette consumption. Similarly, the industry could be held responsible for environmental impacts associated with the sales of their product. In addition, although the tobacco industry has yet to produce a commercially viable biodegradable filter, it may be that there is a technological solution which has so far not met economic requirements. Litigation may change that equation.

Litigation has been pursued against manufacturers of products that damage the environment. In fact, entire communities have filed class action lawsuits to sue polluters, and these cases are typically based on two tort theories: negligence and nuisance. Negligence is a tort theory that permits someone who is injured by another’s unreasonable conduct to recover money damages. The primary element of a successful negligence case is proof of the defendant’s wrongful conduct, or failure to take reasonable steps to prevent the harm. Nuisance is a tort theory that protects someone’s right to use and enjoyment of their real property [41]. Settlement of these cases sometimes requires abatement as well as restitution. Interesting to note is that the responsibility of hazardous waste abatement may include the waste generator who is in part responsible for the waste handler’s actions. Thus, if the handler does a poor job and pollutes the environment, the generator may be responsible for cleanup. One could imagine beach communities in particular resorting to litigation to hold accountable the waste generator (in this case the cigarette manufacturers) for the action of the waste handler (the smoker).

### 5.5. Fines

Fines are levied by local communities for violations of smoking bans on beaches and in enclosed places. Fines for littering may be as high as $1,000 in some states if the littering subject can be observed and cited by authorities. Fines could also be levied by states (or municipalities) against cigarette manufacturers based on the amount of cigarette waste found either as litter or as properly disposed waste. These fines would at least partially compensate for the costs of cleaning up and disposing of cigarette waste; they would certainly be passed along to consumers, thus increasing the costs of smoking and reducing consumption.

### 5.6. Mandatory Filter Biodegradability

Food and Drug Administration (FDA) Regulation of Tobacco products is now being considered for authorization under the US Senate *Family Smoking Prevention and Tobacco Control Act* (already passed by the House of Representatives and not approved in the Senate). If passed, this act would:

- Empower the FDA to establish a periodically re-evaluated content standard, and require changes in tobacco products to meet the standard.
- Grant the FDA authority to require changes in current and future tobacco products to protect public health, such as the reduction or elimination of harmful ingredients, additives and constituents, including smoke constituents.
- Empower the FDA to reduce nicotine yields to any level other than zero (reserved to Congress). This means the FDA can reduce nicotine to minimal levels, including levels that do not lead to addiction.
- Authorize the FDA to require the reduction or removal of harmful or potentially harmful constituents to protect the public health [42].

Clearly, this legislation would have implications for states that hope to regulate tobacco products in any way, and there is concern among tobacco control advocates as to whether such regulation would pre-empt state actions. However, there is already precedent for state regulation of tobacco projects. Cigarettes are regulated by 22 states to be fire safe if sold in a specific state. Canada has become the first nation to mandate the sale of fire-safe cigarettes [43]. State legislation to mitigate a significant non-point-source of environmental pollution may be an effective means of either prohibiting the sale of cellulose-acetate filtered cigarettes or mandating that only biodegradable filtered cigarettes could be sold in the state.

### 5.7. Ban Disposable Filters

Some products known to be hazardous or prone to improper disposal have simply been banned entirely from sales and distribution. For example, pop-tops on aluminum cans [44], which were frequently littered and caused injury when stepped on, and plastic tampon applicators, which even when disposed of properly tended to wash up on beaches [45] were regulated by state laws. Thus, States could simply ban the sale of filtered cigarettes if these were to be considered as an environmental problem. This controversial proposal requires further research to determine its potential individual and population health impacts. There may in fact be significant positive behavioral impacts in reducing smoker’s consumption of unfiltered cigarettes or reducing initiation among children.

### 5.8. Consumer Education and Responsibility

There are several grass roots organizations and websites addressing the issue of cigarette butt waste, both in the United States and elsewhere around the world (Table 2). These focus primarily on consumer education and responsibility to dispose of butts properly. Many, such as KAB, may be funded by the tobacco industry [46]. However, it is an accepted notion in health behavior science that human behavior changes only slowly if at all unless there are costs, benefits, and social norms to support these changes. Butt littering is for the most part an ignored behavior among smokers; it may even be a part of the smoking ritual. Added to this is the now widespread regulation of indoor smoking, which causes smokers to retreat to the street and sidewalk where there may be no butt receptacles. The question arises as to the responsibility to provide suitable receptacles. Should these be the property owner, the city or county, or should there be requirements for all smokers to carry hand-held ashtrays? If they did carry and use these, how would disposal of the ashtray contents be regulated or assured?

Public information campaigns that involve all stakeholders will be important no matter what the policy approaches to controlling butt waste. Public enforcement of littering regulations will follow changing social norms. Increased regulatory activity at the state and local level will follow raised awareness of the butt litter problem. Increased publicity about ‘green’ behavior may affect the littering behavior of smokers. Added to this are fines, fees, and other economic disincentives, and smokers may change behavior even more. One thing is certain, however: when cigarette consumption decreases as a result of reduced prevalence of smoking, butt waste decreases. In the last ten years, the per capita consumption of cigarettes declined almost 20% in the United States [4].

## 6. Discussion

Cigarette butts are undoubtedly an environmental problem causing blight on beaches, streets, sidewalks, waterways, and public spaces. Most of the policy approaches proposed above would likely have two benefits to health and the environment. First, they would likely increase the costs of cigarettes to consumers, as manufacturers would pass along the costs of taxes, fees, litigation, or new production technology. Increasing the price of smoking is a well-established way to reduce smoking [47]. Even a returnable deposit, if large enough, might deter some from starting to smoke, since it would require a larger initial outlay. Reduced smoking rates would in turn lead to fewer discarded butts. The health consequences of changing or removing filters from the market altogether are not known. However, the possibilities range from improved population health due to decreased consumption (if smokers were induced to quit by the absence of their preferred cigarettes, and the loss of the psychological “safety” of filters); worse population health (if smokers continued to smoke unfiltered, somewhat more hazardous cigarettes); or unchanged population health (if new products created in response to these regulations replaced filtered cigarettes, or if filters are confirmed to have no appreciable health benefits). New products might include cigarettes with toxins removed in some other way, or the introduction of non-disposable, reusable filters. Under the new FDA regulations that may be authorized by Congress, changes in the tobacco products would need to undergo FDA review.

Second, adoption of these policies would mean no longer allowing the industry to externalize the costs of the cleanup of butt litter. The current industry approach (as with its historical approach to the direct health consequences of smoking) is basically to ‘blame the victim’. In this context, the smoker is the litterer and thus it is his or her responsibility to take care of the butt disposal. However, it is clear that municipalities, businesses, states, voluntary groups, and other external bodies bear the brunt of most butt waste cleanup costs.

Although some aspects of tobacco product policy in the United States are reserved for the Federal government (for example, labeling), others are clearly in the camp of state or local intervention. For example, states are increasingly requiring that cigarettes sold be designed for Reduced Ignition Propensity (RIP), to reduce fire risk. Pollution mitigation fees can be charged at numerous governmental levels. It is clear that under current conditions Federal authority is not required to adopt state or local policies aimed at reducing cigarette litter and waste.

There may be drawbacks or unintended consequences to many the policies to control butt waste. Would biodegradable filters make smoking more acceptable, or allow cigarette companies to tout their products as “green”? Would states or municipalities come to rely on taxes, fines, or fees, and therefore be reluctant to impose new tobacco control laws that might reduce revenue? Would the negative health consequences of banning or changing filters outweigh the behavioral changes anticipated in removing them from the market? Clearly, more research is called for on many of these issues, especially on the behavioral effects on smokers and potential smokers, and on the economic impact of butt waste cleanup.

## Figures and Tables

**Table 1.** Smoking bans on beaches by State and Municipality, United States, 2008.

**State**	**Municipality**
California	Albany, Belmont, Calabasas, Capitola, Carmel, Carpinteria, Del Mar, El Cajon, El Segundo, Encinitas, Hayward, Hermosa Beach, Imperial Beach, Laguna Beach, Loma Linda, Los Angeles, Los Angeles County, Manhattan Beach, Monterey, Morro Bay, Novato, Oceanside, Pacific Grove, Pacifica, Palos Verdes Estates, San Diego, San Mateo County, Sand City, Santa Cruz, Santa Monica, Seal Beach, Torrance
Florida	Jupiter Island
Hawaii	Hawaii County
Iowa	Des Moines, Johnson County
Illinois	Chicago, Highland Park, Lake Forest, Wilmette
Massachusetts	Abington, Braintree, Grafton, Holliston, Sharon, Tyngsborough, Upton, Westford
Michigan	Grand Haven Township, Howell, Ottawa County
Minnesota	Battle Lake, Bloomington, Buffalo, Fergus Falls, Hennepin County, Hoffman, Ramsey County, Washington County
New Hampshire	Gilford, Windham
New Jersey	Brick Township, Dover Township, Lavallette Borough, Mount Arlington Borough, Seaside Park, Ship Bottom Borough, Surf City Borough
New York	Kingston
Puerto Rico	Puerto Rico
Rhode Island	Westerly
South Carolina	Surfside Beach
Utah	Davis County
Washington	Lake Stevens
Wisconsin	Madison

Source: Personal communication, B. Frick, Americans for Nonsmokers Rights, December 2008

**Table 2.** Environmental Groups Concerned with Cigarette Butt Waste.

**Organization**	**Main Focus**	**Website**
Surfrider Foundation	Clean Water, Beach Access, Beach Preservation and Protecting Special Places	http://www.surfrider.org/a-z/cig_but.php
Earth Resource Foundation	Environmental Education	http://www.earthresource.org/events/hotyb-current.html
Clean Virginia Waterways	Waterway cleanup	http://www.longwood.edu/cleanva/cigarettelitterhome.html
Ocean Conservancy	International Coastal Cleanup	http://www.oceanconservancy.org/site/PageServer?pagename=icc_home
Queensland Litter Prevention Alliance	Anti-litter advocacy	http://www.qldlitter.com/litter_facts.php
ButtsOut	Personal Ashtrays	http://www.buttsout.net/UK
